# Contingency Management for Dually Diagnosed Inpatients with Psychosis

**DOI:** 10.3390/ijerph21050578

**Published:** 2024-05-01

**Authors:** Lise Docx, Marianne Destoop, Geert Dom

**Affiliations:** 1 Multiversum, 2530 Boechout, Belgiumgeert.dom@uantwerpen.be (G.D.); 2 Faculty of Medicine, CAPRI, University of Antwerp, 2610 Antwerp, Belgium

**Keywords:** contingency management, psychosis, substance use disorder, inpatient

## Abstract

Contingency management (CM) is an evidence-based treatment method in substance abuse treatment. However, little is known about its efficacy in dually diagnosed patients with psychosis and in inpatient settings. Therefore, the aim of this study is to investigate the efficacy of CM for dually diagnosed patients with psychosis in an inpatient setting. Furthermore, we investigate the effect of the nature of the reward used (cash vs. prize) on the efficacy of CM. We made use of an 8-week fish-bowl CM intervention by means of a within-subject reversal design with three treatment phases (baseline—intervention—follow-up). Sixty-seven patients were included in this study, of whom thirty-four completed the protocol. The results show no effect of CM on abstinence nor an interaction with clinical or demographic variables. Cash money is as effective as prizes. Future research should further investigate the effect of psychosis and treatment setting on the efficacy of CM, with special attention for Patient Report Experience and Outcome Measures (PREM/PROM).

## 1. Introduction

Dually diagnosed patients who suffer from both substance use disorder (SUD) and psychosis are an especially vulnerable patient population. Studies report that about 40 to 50% of patients with a psychotic disorder have a co-occurring SUD [1,2]. These patients have been shown to experience higher levels of psychopathology, especially positive symptoms, experience higher relapse and hospitalization rates, report lower quality of life, display greater disability, and have a younger age at death and higher suicide rates than patients with psychosis who do not experience substance abuse [1,3,4,5,6,7,8,9,10,11]. Continuing substance use seems to be a determining risk factor in this poor outcome. In fact, effective treatment of SUD in patients with psychosis improves outcomes significantly [12,13].

Thus, there is a high need for effective and evidence-based treatment options for SUD for these patients. Contingency management (CM), a behavioral treatment offering reinforcers for behavior in line with treatment goals (in SUD, most often drug-negative urine samples), has shown promising results in general SUD treatment. Numerous meta-analyses have been performed that indicate CM’s superiority to treatment as usual in the treatment of opioid, cocaine, cannabis, nicotine, stimulant, and poly-drug abuse [14,15,16]. Over the years, several CM protocols have been developed, with voucher-based, prize-based, or fishbowl methods being the most implemented [17]. Both methods have been shown to be equally effective as long as some key ingredients are respected [18]. These key ingredients are immediacy, escalating reinforcement schedules, and the appropriate perceived value of rewards [19].

Although studies investigating the effectiveness of CM in dually diagnosed patients with psychosis are scarce, a recent meta-analysis performed by our group indicated that CM is effective, with patients receiving CM being more likely to be abstinent. The effect sizes, however, are lower than those generally found in addiction research. In contrast to general SUD evidence, there was no effect on retention in treatment [20]. These findings are in line with another recent review, showing CM to be efficacious in producing cannabis use reductions and abstinence amongst individuals with a psychotic spectrum or major depressive disorder [21].

In the present study, we aim to elaborate our knowledge on the use of CM in dually diagnosed patients with psychosis by focusing on both the effect of the context in which the intervention is implemented and on the nature of the reward. Most studies on CM thus far have been performed in outpatient treatment settings, and little is known about its efficacy in inpatient settings. Although the intervention as such can be easily implemented in an inpatient setting, it is possible that the context of hospitalization has an effect on its efficacy (e.g., fewer substance use triggers, high availability of staff members, group dynamics). Therefore, we implemented a fishbowl CM intervention on an inpatient treatment ward for dually diagnosed patients with psychosis in Belgium. To examine the effect of the nature of the reward (cash or exchangeable vouchers), participants were randomized into two groups. Most CM interventions make use of vouchers that can be exchanged for goods or services; however, Festinger et al. [22] suggest that making use of cash money is equally effective and is preferred by service users. Furthermore, offering cash money is a way of making the intervention more cost-effective, as it saves on staff members needed to obtain and exchange the rewards.

We hypothesize that CM leads to fewer drug-positive urine samples, independent of reward nature.

## 2. Materials and Methods

### 2.1. Participants

Participants were recruited on ‘Cadenza 4A’ of the psychiatric hospital Multiversum, Boechout, Belgium. This is an inpatient treatment ward for patients with a dual diagnosis of substance use disorder and psychosis. Patients were hospitalized but were allowed to leave the facility in the evenings and weekends, with a maximum of 1 overnight stay outside the facility each week. The hospital protocol prescribed that if patients tested positive for any substance, they were not allowed to leave the facility for 7 days. The standard treatment patients received was an integrated treatment consisting of a combination of psychopharmacology, individual and group cognitive-behavioral therapy, with a focus on motivational interviewing, relapse prevention, and systemic interventions.

All patients who were thought eligible for the study, based upon a diagnostic interview with a psychiatrist, were asked to participate by a bachelor degree study nurse and were included after providing written informed consent. The study was conducted in accordance with the Declaration of Helsinki, and the protocol was approved by the Ethics Committee of the University Hospital of Antwerp (UZA) (project identification code 20/12/137).

In total, 67 participants were included in the study (of whom 34 completed the study).

After inclusion, patients were randomized into two groups: a group that was rewarded with cash money and a group rewarded with exchangeable vouchers. There was no difference between the groups on clinical or demographic variables, except for the ‘personality’ score on the MATE (t = 2.731, *p* = 0.008, d = 0.707). In the group of completers, we found similar results, with the MATE ‘personality’ score being the only measure that differed significantly (U = 53.00, *p* = 0.008, r = 0.468).

Demographic and clinical variables of both included patients as completers are listed in Table 1.

### 2.2. Materials

#### 2.2.1. Brief Psychiatric Rating Scale (BPRS) 

The BPRS [23] is a semi-structured interview aimed at assessing the severity of general psychiatric symptoms. It consists of 18 items, rated on a scale from 1 (not present) to 7 (extremely severe).

#### 2.2.2. Measurements in the Addictions for Triage and Evaluation (MATE 2.1) 

The MATE [24] is a semi-structured interview that is developed to identify patient characteristics in addiction treatment. It results in 20 scores: characteristics of physical comorbidity; undergoing psychiatric or psychological treatment; characteristics of psychiatric comorbidity; dependence; abuse; severity of dependence/abuse; physical complaints; personality (a screening for personality disorders); limitations—total; limitations—basic; limitations—relationships; positive external influences; negative external influences; care and support; need for care; craving; depression; anxiety; stress; and depression anxiety stress—total.

#### 2.2.3. Contingency Management Intervention

We made use of a fishbowl CM intervention [25]. Each fishbowl had 500 fiches, of which 250 were social reward (“well done”, “good job”, …, etc.); 174 had a value of EUR 2; 50 had a value of EUR 5; 25 had a value of EUR 20; and 1 was a jumbo prize of EUR 100. Urine samples were collected twice a week, and in the active treatment phase, negative samples were rewarded in line with the following scheme: after a first negative urine sample, a participant was entitled to 1 draw, after the second negative sample, 2 draws, etc. The maximum number of draws was 8, so if patients stayed abstinent after their 8th draw, they continued to draw 8 fiches. Following a positive urine sample, no reward possibility followed, and the reward schedule was reset to 1 draw.

Rewards were either cash money or vouchers that could be redeemed for goods available on the treatment ward.

Only the results of the main product of abuse were taken into account for the CM intervention (i.e., if cannabis was the main product of abuse and the participant tested positive for amphetamines, he/she did receive a reward).

### 2.3. Protocol

We made use of a within-subject reversal design consisting of 3 phases: baseline, active intervention, and follow-up.

#### 2.3.1. Baseline (0–4 Weeks)

In the first 4 weeks of the study, patients were interviewed by the study nurse using the BPRS and MATE 2.1. The results of the MATE were used to identify the main product of abuse and were in agreement with the patient. Participants were randomized to the cash or voucher group by means of the randomization software (Randomness and Integrity Services LTD, Dublin, Ireland) (https://www.random.org/, accessed on 1 April 2019).

Monitoring of drug use was started in this baseline phase by means of urine toxicology twice a week. In this study phase, no reward was provided for clean urine samples.

#### 2.3.2. Active Intervention (4–12 Weeks)

In the active intervention phase, CM was provided for 8 weeks by means of the fishbowl method described above. At the start of the intervention phase, participants were offered a priming session in which they could draw fiches from their fishbowl until they obtained a tangible reward.

For further analysis, this phase was divided into two 4-week periods in order to equally compare time frames.

#### 2.3.3. Follow-Up (12–16 Weeks)

After the active intervention phase ended, drug use was monitored for another 4 weeks use with twice-weekly urine samples.

### 2.4. Data Analysis

The results were analyzed using IBM SPSS Statistics 29.0

To evaluate the randomization across groups, clinical and demographic variables of the money and voucher groups were compared by means of independent sample *t*-tests (at inclusions) and Mann–Whitney tests (for completers).

The effect of CM was analyzed with a repeated-measures ANOVA with the percentage of positive urine samples in the different treatment phases (baseline—week 5 to 8—week 9 to 12—follow-up) as the dependent variable.

To analyze the effect of reward type, we performed a Kruskal–Wallis test with the percentage of positive urine samples in the different treatment phases as the dependent variable and reward type as the grouping variable.

In order to investigate the effect of possible moderating variables, the severity of the addiction index of MATE 2.1, the main product of abuse and patients’ legal situations were used as grouping variables in second-order analyses.

Clinical measures of patients who completed the study and those who dropped out were compared by means of independent sample *t*-tests.

The significance level was set at *p* < 0.05. Since this was an exploratory study, we opted not to correct for multiple comparisons.

## 3. Results

### 3.1. Dropout

About half of the included participants completed the study (34 out of 67). Of the participants who dropped out, 14 dropped out in the first phase of the study, where drug use was monitored without reinforcement; 9 dropped out in the first 4 weeks of the intervention; 2 dropped out in the second phase of the intervention; and 8 dropped out in the follow-up phase of the study. Patients dropped out of the study because they were discharged from the hospital. In eight cases, discharge was demanded by the treatment team because of serious violation of treatment rules (e.g., use of substances on campus); in nine cases, patients requested their discharge themselves; in twelve, cases discharge was in mutual agreement between the treatment team and patient. For one patient, the study was ended because of the use of ketamine, a drug that we were not able to monitor adequately in line with CM guidelines.

Patients who completed the study and those who dropped out differed significantly on the following clinical and demographic variables: MATE psychiatric comorbidity (t = 1.926, *p* = 0.029, d = 0.490), MATE physical complaints (t = 2.273, *p* = 0.014, d = 0.591), MATE care and support (t = 1.950, *p* = 0.028, d = 0.501), and MATE craving (t = 2.556, *p* = 0.007, d = 0.696), with those with higher levels of symptoms being at a higher chance to drop out of the treatment.

For an overview of the mean MATE index scores for those who completed the study and those who dropped out, see Table 2.

### 3.2. Contingency Management

**Figure 1:**
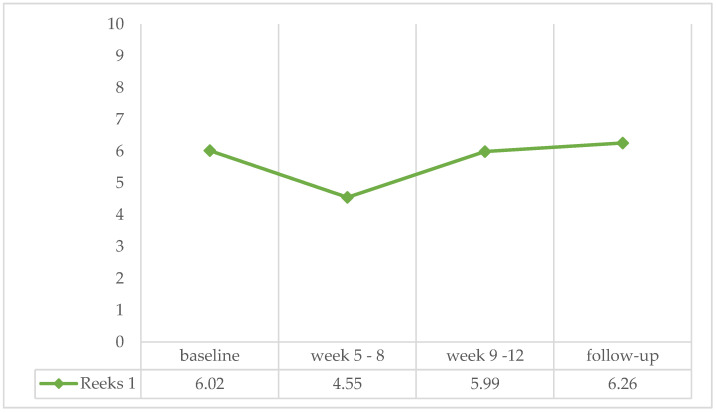
Percentage of positive urine samples (US) over the four treatment phases.

The results show no effect of treatment phase (F(3) = 0.207, ns, η^2^ = 0.006). This means that the percentage of positive urine samples was the same across the treatment phase (see Figure 1). The results stayed the same when analyses were performed on the group who dropped out during follow-up (F(2) = 0.390, ns, η^2^ = 0.012) or during treatment phase 2 (F(1) = 0.730, ns, η^2^ = 0.022) (see Figure 1).

**Figure 2:**
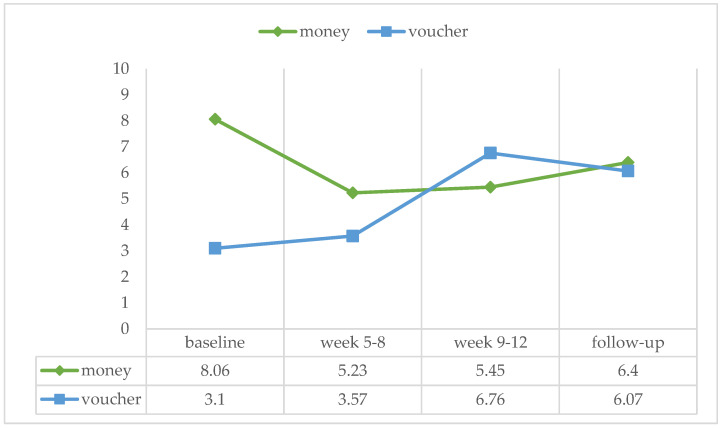
Percentage of positive urine sample over the four treatment phases for the money (*n* = 20) and voucher group (*n* = 14).

We also could not find an effect of the reward type on the mean percentage of positive urine samples in any of the treatment phases (baseline: U = 117.5, ns, r = −0.168; week 5–8: U = 133.00, ns, r = −0.056; week 9–12: U = 134.50, ns, r = −0.038; follow-up: U = 125.5, ns, r = −0.063) (see Figure 2).

### 3.3. Effect of Severity of Addiction

**Figure 3:**
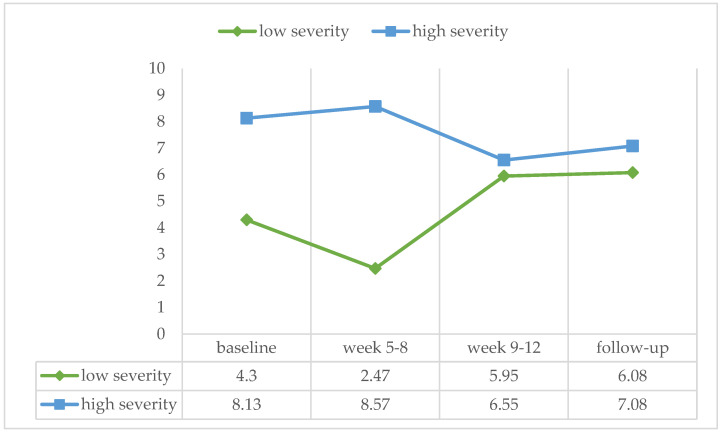
Percentage of positive urine samples over the four treatment phases for patients scoring low (*n* = 21) and high (*n* = 12) on the severity of addiction index of the MATE 2.1.

According to the MATE algorithm, 12 patients were categorized with a high severity of addiction. A Mann–Whitney test for independent samples showed no significant difference between groups in any of the treatment phases (baseline: U = 108.50, ns, r = −0.145; week 5–8: U = 92.00, ns, r = −0.295; week 9–12: U = 116.50, ns, r = −0.071; follow-up: U = 119.00, ns, r = −0.009) (see Figure 3).

### 3.4. Effect of Diagnosis

**Figure 4:**
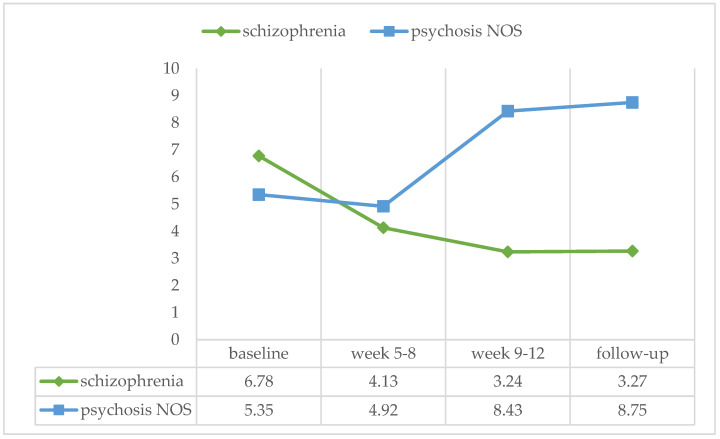
Percentage of positive urine samples over the four treatment phases for patients with schizophrenia/schizoaffective disorder (*n* = 16) and psychotic disorder NOS (not otherwise specified) (*n* = 18).

Sixteen patients were diagnosed with schizophrenia/schizoaffective disorder, and eighteen patients with psychotic disorder NOS. A Mann–Whitney test for independent samples showed no significant effect of diagnosis in any of the treatment phases (baseline: U = 136.00, ns, r = −0.059; week 5–8: U = 140.00, ns, r = −0.032; week 9–12: U = 100.00, ns, r = −0.299; follow-up: U = 124.5, ns, r = −0.088) (see Figure 4).

### 3.5. Effect of Legal Situation

**Figure 5:**
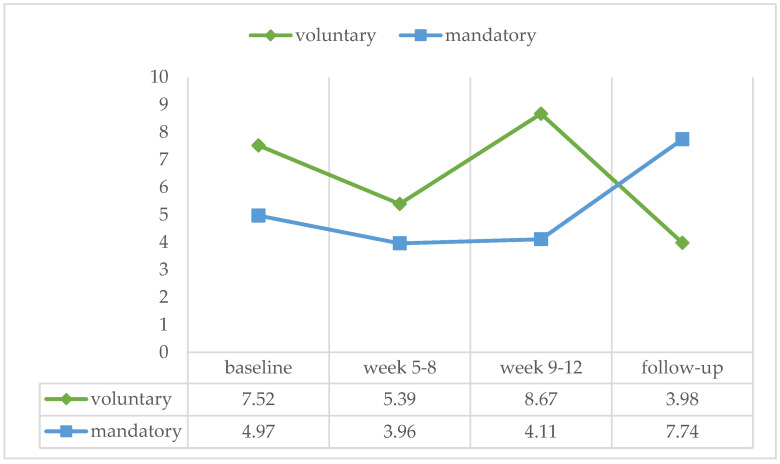
Percentage of positive urine samples over the four treatment phases for patients being in treatment voluntary (*n* = 14) or mandatory (*n* = 20).

In this study, patients who were in voluntary treatment as well as patients who were admitted as mandatory were included. This had no effects on the intervention efficacy (baseline: U = 125.50, ns, r = −0.108; week 5–8: U = 128.00, ns, r = −0.097; week 9–12: U = 96.00, ns, r = −0.202; follow-up: U = 121.50, ns, r = −0.073) (see Figure 5).

## 4. Discussion

In contrast to our hypothesis, we could not find an effect of CM on drug use in an inpatient sample of dually diagnosed patients with psychosis. This effect was not moderated by the nature of the reward, the severity of addiction, or the legal situation.

These results are in contrast with the existing evidence. Several hypotheses can be taken into account to explain these unexpected findings.

First, the present study was performed in an inpatient setting, whereas most studies so far have been performed in outpatient treatment clinics. It is possible that the effect of being in a structured and safe environment during hospitalization minimizes the possible additive effect of a CM intervention. Across all study phases, very low percentages of drug-positive urine samples are found, pointing to a possible ceiling effect previously described by Hagedorn et al. [26] and Petry et al. [27], who report that if drug use is low, the additive effect of CM seems negligible. However, the absent effect of CM on abstinence stands in sharp contrast to the reported experiences of staff members and included patients. Both groups describe overtly positive effects of the implementation of CM on treatment engagement and ward atmosphere (e.g., willingness to try to obtain abstinence, levels of incidents between staff and patients, on site drug use, quality of therapeutic relationship, …, etc.). These staff and patient feedbacks are in line with recent qualitative studies showing that CM is seen as a facilitator of extended engagement in treatment, and an encouragement for clients to make progress in the treatment process [28,29]. Given the lack of a qualitative part in our study protocol, we cannot objectify these experiences, but it does hint at the possibility that abstinence might not be the most suitable outcome measure in this setting. In line with the growing awareness that highlights the importance of Patient Reported Experience Measures and Patient Reported Outcome Measures (PREMS and PROMS) [30], it would be interesting to focus on PREMS/PROMS and the experiences of staff members in future research.

Another possible explanation lies in the investigated patient population. Although a recent meta-analysis performed by our group did find a positive effect of CM in patients with psychosis, the effect size is lower than that reported in general addiction research [20]. Thus, it might be the case that CM is less effective for patients suffering from psychosis. A possible explanation for this hypothesis can be found in the described deficits in the reward system of patients with psychosis. Although immediacy of reward is one of the key aspects of CM, a certain amount of time delay between withholding from drug use and reward is inevitable (i.e., patients do not have the option to choose for the reward in the moment possible drug use emerges). Thus, it might be that the representational deficits in reward processing reported in patients with psychosis make CM less effective in this population [31].

In line with our expectations, the nature of the reward had no impact on the results. This is an interesting finding, as cost-effectiveness is one of the most reported thresholds for implementing CM in clinical practice. If cash rewards appear to be as effective and safe as vouchers or prizes, this might entail a significant cost reduction in the implementation of CM, as the staff costs of obtaining and exchanging prizes are minimized. Furthermore, Festinger et al. [22] report that cash is also preferred by service users, which might help motivate patients to engage in treatment.

Some limitations must also be taken into account when interpreting the results.

First, the within-subject reversal design was highly effective for investigating the feasibility of implementing a CM protocol on an inpatient ward, but to investigate its efficacy, a randomized control trial would have been more suitable.

The choice for a more naturalistic approach also resulted in a sample that was predominantly male, which is in accordance with the patient population treated at the investigated ward but also with what is reported in the literature (e.g., [2,32]). It might be of interest to investigate if men and women respond differently to CM, although a systematic review by Forster et al. [33] suggests that sex does not have an effect on the efficacy of CM. Another consequence of this choice was the diagnostic heterogeneity of the sample. Both patients with schizophrenia or schizoaffective disorder and patients with the psychotic disorder NOS were included. Although we could not find an impact of the diagnostic group on the results in our current sample, it would be interesting to see in future studies if these diagnostic groups might react differently to CM. Furthermore, the main product of abuse varied across patients. Although CM has been proven effective in the treatment of stimulant, cocaine, cannabis, and opioids abuse [14,15,16], it could be that in dually diagnosed patients, the product of abuse does impact the efficacy of CM. Given the small sample size, the current study does not allow for such comparisons.

Secondly, high dropout rates had a negative effect on the statistical power of the study. In combination with the low number of positive urine samples (and thus much lower possible effect sizes than those reported in the literature), this might have impacted the results. This warrants caution when interpreting the results, as the negative results might be related to the sample size. Furthermore, the high dropout rates limit the generalizability of the results. Of interest in this account is that patients who dropped out of the study had higher symptom levels on some MATE indices. Thus, it might be that those who could potentially benefit the most from the intervention (i.e., research indicates that those with more severe substance abuse benefit more from CM) were more likely to drop out. Furthermore, it might be that patients with higher symptom levels experience greater difficulties following hospital regulations (e.g., no onsite drug use) and therefore have a higher chance of dropping out of treatment. Petry et al. [27] suggested that patients with more severe substance use disorders might need a higher magnitude of CM. We minimized the length of the CM intervention to 8 weeks (the minimal duration to obtain the effect described) in order to minimize dropout, but this might have had a reverse effect.

Finally, the magnitude of the reward has been reported to be an important factor in determining the efficacy of CM. Although the expected average amount of EUR 240 in this study has been found to be effective [27], it is possible that for those with more severe SUD magnitudes, it needs to be higher to improve outcome and/or treatment retention.

## 5. Conclusions

In conclusion, in this within-subject reversal study, we could not find an effect of CM on abstinence in a sample of dually diagnosed inpatients with psychosis. Future research should investigate the efficacy of CM in inpatient settings and in patients with psychosis further, taking into account a broader range of outcome measures than abstinence.

## Figures and Tables

**Table 1:** Demographic and clinical variables.

	Inclusion		Complete	
	Money (37)	Voucher (30)	Money (20)	Voucher (14)
Age (years)	36.33 (13.94)	34.38 (7.90)	33.45 (6.88)	33.71 (10.18)
Male/female	30/7	23/7	18/2	11/3
Mandatory/voluntary	26/21	15/15	10/10	10/4
Diagnosis				
Schizophrenia or schizoaffective disorder	17	11	9	4
Psychotic disorder NOS	20	19	11	10
Main product of abuse				
- Amphetamines	9	9	7	2
- Cannabis	15	11	9	8
- Cocaine	11	7	4	2
- Opioides	0	2	0	1
- Benzodiazepines	2	1	0	1
Dropout (male/female)	17 (12/5)	16 (12/4)	_	_
BPRS	35.83 (7.82)	37.07 (12.55)	36.45 (12.56)	33.38 (6.81)
DOT (days)	63.76 (122.10)	50.73 (78.71)	79.65 (148.60)	60.43 (61.11)
MATE				
Physical comorbidity (0–4)	0.31 (0.68)	0.37 (0.74)	0.20 (0.52)	0.31 (0.63)
Psychiatric/psychological treatment (0–2)	1.86 (0.46)	1.85 (0.46)	1.80 (0.41)	1.77 (0.60)
Psychiatric comorbidity (0–5)	1 (1.11)	1.04 (1.22)	0.95 (1.10)	0.46 (0.66)
Dependence (0–7)	4.89 (2.13)	4.41 (2.26)	4.65 (2.13)	3.77 (2.49)
Abuse (0–4)	2.31 (1.30)	2.22 (1.28)	2.45 (1.32)	2.15 (1.34)
Severity of dependence/abuse (0–9)	6.17 (2.46)	5.56 (2.87)	6.05 (2.44)	4.85 (3.31)
Physical complaints (0–40)	10.37 (9.17)	7.85 (6.30)	8.05 (7.65)	5.50 (4.40)
Personality (0–8)	**4.29 (2.04)**	**3.00 (1.47)**	**4.25 (1.83)**	**2.58 (1.00)**
Limitations—total (0–76)	15.23 (13.39)	12.67 (9.91)	13.05 (13.59)	11.69 (10.70)
Limitations—basic (0–32)	4.80 (5.32)	3.96 (4.85)	3.7 (5.32)	3.54 (5.61)
Limitations relationships (0–20)	4.06 (4.11)	3.48 (3.50)	4.20 (4.48)	3.15 (4.10)
Care and support (0–32)	4.94 (4.28)	6.15 (3.58)	4.50 (4.52)	4.69 (3.75)
Positive external influences (0–12)	4.62 (2.67)	4.85 (3.50)	4.30 (2.03)	4.61 (2.06)
Negative external influences (0–20)	3.53 (3.16)	3.44 (2.15)	3.45 (3.33)	3.00 (1.91)
Need for care (0–20)	2.13 (4.32)	1.63 (2.63)	2.00 (4.53)	1.67 (3.61)
Craving (0–20)	5.45 (4.28)	5.65 (4.17)	4.06 (3.98)	4.30 (3.02)
Depression (0–42)	8.90 (8.61)	8.87 (7.05)	8.76 (9.17)	6.80 (6.55)
Anxiety (0–42)	8.58 (9.36)	8.35 (6.49)	8.94 (10.73)	5.60 (5.56)
Stress (0–42)	8.13 (8.98)	8.43 (8.51)	9.06 (10.87)	4.80 (6.12)
Depression anxiety stress—total (0–126)	24.35 (24.28)	25.65 (19.64)	24.82 (29.03)	17.20 (16.84)

BPRS = Brief Psychiatric Ratings Scale; DOT = Duration of Treatment; MATE = Measurements in the Addictions for Triage and Evaluation. Bold scores differ significantly.

**Table 2:** Mean MATE index scores for completers (*n* = 33) and patients who dropped out (*n* = 34). Bold scores differ significantly.

	Complete	Dropout
Physical comorbidity	0.24	0.45
Psychiatric/psychological treatment	1.79	1.93
Psychiatric comorbidity	**0.76**	**1.31**
Dependence	4.3	5.1
Abuse	2.33	2.21
Severity of dependence/abuse	5.58	6.28
Physical complaints	**7.09**	**11.72**
Personality	3.63	3.86
Limitations—total	12.51	15.93
Limitations—basic	3.64	5.34
Limitations—relationships	3.8	3.83
Care and support	**4.58**	**6.54**
Positive external influences	4.42	5.07
Negative external influences	3.27	3.75
Need for care	1.89	1.95
Craving	**4.15**	**6.93**
Depression	8.04	9.74
Anxiety	7.7	9.26
Stress	7.48	9.04
Depression anxiety stress—total	22	27.81

## Data Availability

Data are not publicly accessible due to privacy and ethical reasons. Data are available upon request from the corresponding author.
